# Resilience and Daily Mental Health in Hispanic and Latino Dementia Caregivers: A Daily Diary Study

**DOI:** 10.1093/geroni/igaf122.571

**Published:** 2025-12-31

**Authors:** Frank Puga, Natashia Bibriescas, Loreli Alvarez, Stephanie DiFiglia, Andres Azuero, Michael Crowe

**Affiliations:** University of Alabama at Birmingham, Birmingham, Alabama, United States; University of Alabama at Birmingham, Birmingham, Alabama, United States; The University of Alabama at Birmingham, Birmingham, Alabama, United States; Metropolitan Jewish Health System, Inc. Institute for Innovation in Palliative Care, New York, New York, United States; University of Alabama at Birmingham, Birmingham, Alabama, United States; University of Alabama at Birmingham, Birmingham, Alabama, United States

## Abstract

Hispanic and Latino (H&L) family dementia caregivers experience high levels of stress, increasing their risk for depression and anxiety. However, caregiver resilience may mitigate these risks. We examined associations between daily caregiving stress, psychological distress, and resilience using data from the Nuestros Dias (Our Days) Study. Participants (N = 155; 2,679 daily observations) completed daily diary surveys for 21 days, reporting on caregiving stress, depression, and anxiety symptoms using the Neuropsychiatric Inventory–Questionnaire distress ratings and the PROMIS Short Form Depression and Anxiety measures adapted for daily diaries. Resilience factors (perception of self, planned future, and structured style) were measured at baseline using the Resilience Scale for Adults. Data were analyzed using multilevel logistic regression models. On days when caregivers reported higher-than-average caregiving stress, daily odds increased by 7% for depression (OR = 1.07, 95% CI: 1.05–1.09, p < .001) and 9% for anxiety (OR = 1.09, 95% CI: 1.06–1.11, p < .001). For each one-point increase in planned future resilience, daily odds decreased by 25% for depression (OR = 0.75, 95% CI: 0.69–0.83, p < .001) and 21% for anxiety (OR = 0.79, 95% CI: 0.72–0.86, p < .001). Combined effects were examined using mean predicted probabilities based on dichotomized stress and resilience levels. Low resilience/high stress probabilities were 43% and 59% for depression and anxiety, respectively. For high-resilience/high-stress caregivers, probabilities were 14% and 28%, respectively. Findings highlight the need for culturally responsive interventions that foster planned future resilience to enhance H&L caregiver well-being.

